# Development and Temporal Validation of Staged Prediction Models for Acute Respiratory Distress Syndrome After Thymic Epithelial Tumour Resection

**DOI:** 10.3390/jcm15197715

**Published:** 2026-10-06

**Authors:** Ruize Wang, Siyu Yao, Lifeng Huang, Qiuyue Liu

**Affiliations:** 1 Department of Critical Care Medicine, Beijing Tuberculosis and Thoracic Tumor Research Institute, Beijing Chest Hospital, Capital Medical University, Beijing 101149, China; wangrz68@mail.ccmu.edu.cn (R.W.); tpofo0919@stu.xjtu.edu.cn (S.Y.); 2 Intensive Care Unit, Beijing Chao-Yang Hospital, Capital Medical University, Beijing 100020, China

**Keywords:** acute respiratory distress syndrome, thymic epithelial tumours, perioperative care, risk prediction, temporal validation

## Abstract

**Background/Objectives:** To develop and temporally validate staged models for predicting acute respiratory distress syndrome (ARDS) after thymic epithelial tumour resection. **Methods:** This single-centre retrospective study included 596 adults with pathologically confirmed thymic epithelial tumours. Patients treated in 2005–2020 formed the development cohort (*n* = 395), and those treated in 2021–2026 formed the temporal validation cohort (*n* = 201). Postoperative ARDS was adjudicated using the 2024 global definition. A preoperative model and a postoperative landmark model incorporating intraoperative, pathological, and early postoperative variables were developed using least absolute shrinkage and selection operator and multivariable logistic regression. Performance was assessed using discrimination, calibration, Brier score, decision curve analysis, bootstrap internal validation, and temporal validation. **Results:** ARDS occurred in 116 development patients (29.4%) and 58 validation patients (28.9%). The postoperative landmark model included the preoperative linear predictor, one-lung ventilation duration, Masaoka–Koga stage, perioperative transfusion, and early postoperative glucose, albumin, and C-reactive protein. It achieved higher AUCs than the preoperative model in the development (0.908 vs. 0.693; *p* < 0.001) and validation cohorts (0.928 vs. 0.655; *p* < 0.001), with lower Brier scores (0.107 vs. 0.187 and 0.094 vs. 0.192, respectively; both *p* < 0.001). The optimism-corrected development AUC was 0.898. Calibration and decision curve analyses also favoured the postoperative landmark model. **Conclusions:** Incorporating perioperative information improved ARDS prediction after thymic epithelial tumour surgery. The staged models may support sequential perioperative risk assessment; however, prospective evaluation and independent multicentre validation are required before implementation.

## 1. Introduction

Thymic epithelial tumours (TETs), mainly thymoma and thymic carcinoma, are the commonest primary anterior mediastinal neoplasms [1,2], and complete resection remains the principal curative treatment [3,4]. Their proximity to vital structures can necessitate extensive surgery and increase perioperative morbidity [4,5]. ARDS is a life-threatening postoperative pulmonary complication associated with prolonged ventilation and high mortality [6,7].

Several features of patients with TETs may predispose them to postoperative ARDS. TETs frequently coexist with myasthenia gravis (MG), which impairs respiratory muscle strength and airway clearance [5,8]. Locally advanced tumours requiring extended resection, prolonged one-lung ventilation (OLV), or cardiopulmonary bypass impose additional physiological burdens that may promote lung injury [4,9,10].

A recent systematic review identified substantial methodological heterogeneity and frequent risk of bias among ARDS prediction models in adults [11]. Prediction models for postoperative ARDS have been developed in high-risk general surgical populations [12]. Similar models have also been developed and validated in patients undergoing hepatectomy [13] and in patients undergoing cardiac or aortic surgery [14]. In contrast, thymectomy-specific prediction studies have focused on postoperative myasthenic crisis [8] or composite postoperative complications [15], whereas an ARDS-specific model after TET resection with temporal validation remains unavailable.

Because clinically relevant perioperative information accumulates sequentially, preoperative assessment alone may not fully capture risks emerging during or immediately after surgery [16,17]. Accordingly, a preoperative model (Model 1) and a postoperative landmark model incorporating intraoperative and early postoperative information (Model 2) were developed and evaluated in a same-centre temporal validation cohort. An exploratory analysis of factors associated with moderate-to-severe rather than mild ARDS among patients who developed postoperative ARDS was also performed.

## 2. Materials and Methods

### 2.1. Study Design and Participants

This single-centre retrospective observational cohort study used anonymised electronic medical records from Beijing Chest Hospital. Consecutive patients treated from January 2005 to December 2020 formed the development cohort; those treated from January 2021 to April 2026 formed the temporal validation cohort. The Ethics Committee of Beijing Chest Hospital approved the study (approval number: YJS-2025-03) and waived the requirement for individual informed consent given the retrospective observational design and use of anonymised data. This study was conducted in accordance with the Declaration of Helsinki and reported in accordance with the STROBE statement and the TRIPOD + AI reporting guideline [18,19].

Patients were eligible if they met all of the following criteria:

(1) Age ≥ 18 years;

(2) Thymectomy or extended tumour resection;

(3) Pathological confirmation of thymoma or thymic carcinoma according to the 2021 World Health Organization classification [1];

(4) Adequate documentation to establish eligibility, predictor timing, and postoperative ARDS status.

Patients were excluded if any of the following criteria were present:

(1) A concurrent major thoracic or cardiovascular procedure, including pneumonectomy;

(2) Pre-existing ARDS, a preoperative PaO_2_/FiO_2_ ratio < 300 mmHg, or a requirement for preoperative mechanical ventilation;

(3) A final pathological diagnosis other than a TET;

(4) Insufficient data to determine the primary outcome or key predictor timing.

**Figure 1:**
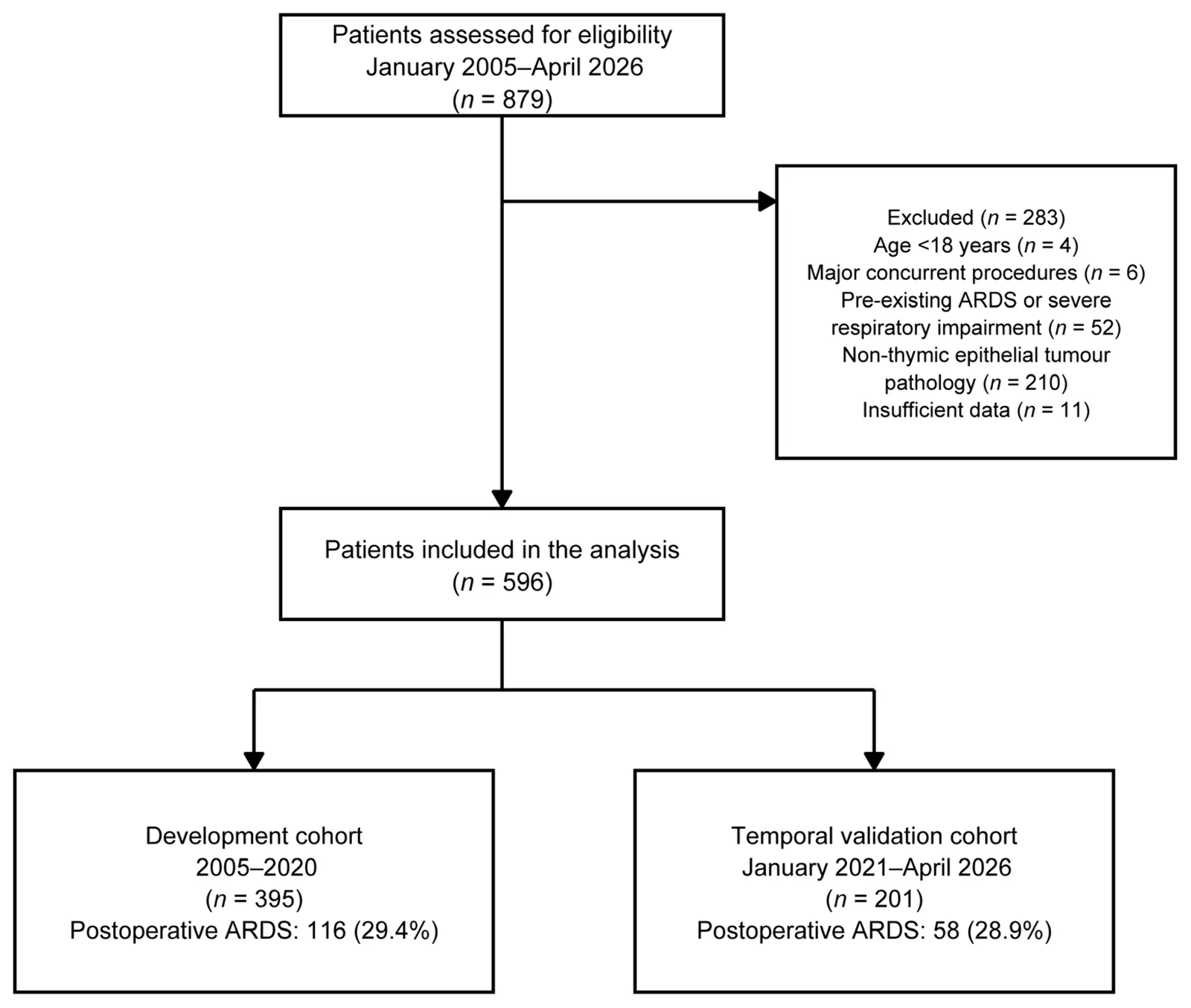
Patient selection flowchart. Of 879 patients assessed, 596 were included, comprising 395 patients in the development cohort and 201 in the temporal validation cohort. Abbreviation: ARDS, acute respiratory distress syndrome.

Patients who met all inclusion criteria and none of the exclusion criteria were included in the study, as shown in Figure 1.

### 2.2. Outcomes

The primary outcome was postoperative ARDS, independently adjudicated according to the 2024 global definition [6]. Diagnosis required: new or worsening respiratory symptoms within 7 days of surgery; bilateral pulmonary opacities not fully explained by pleural effusion, atelectasis, or nodules; respiratory failure not primarily attributable to cardiac failure or fluid overload; and qualifying oxygenation impairment under specified respiratory-support conditions. Severity was stratified according to the lowest qualifying PaO_2_/FiO_2_ ratio (mild: 201–300 mmHg; moderate: 101–200 mmHg; severe: ≤100 mmHg); SpO_2_/FiO_2_ thresholds were used only when arterial blood gas measurements were unavailable and SpO_2_ was ≤97%. Moderate and severe ARDS were combined for the exploratory severity analysis. Outcome discordance between investigators was resolved by consensus or adjudication by a third investigator.

### 2.3. Candidate Predictors

Preoperative predictors encompassed demographics, comorbidities, pulmonary function indices and routine laboratory measurements; selected baseline characteristics are presented in Table 1, with additional variables in Supplementary Table S2. Intraoperative and pathological variables included surgical approach, operative duration, OLV duration, blood loss, tumour diameter, histological type, Masaoka–Koga stage (I–II versus III–IV), and perioperative transfusion. Early postoperative laboratory values—glucose, sodium, potassium, creatinine, albumin, C-reactive protein (CRP), white blood cell count, neutrophil percentage, haemoglobin, and platelet count—were obtained from blood samples drawn approximately 1 h after transfer to the post-anaesthesia care unit or intensive care unit (permissible window, ±30 min) and were required to precede the first clinical ARDS diagnosis.

Masaoka–Koga stage was derived from the final pathology report, which was typically available within the first 3–5 postoperative days. Model 2 was therefore designed as a postoperative landmark model, with the landmark defined as the time at which all included predictors, including the final pathological Masaoka–Koga stage, had become available. The timing of the first documented postoperative ARDS diagnosis was reviewed relative to this landmark. No documented ARDS events occurred before the landmark in either the development or temporal validation cohort. Accordingly, Model 2 estimates ARDS risk after the landmark assessment rather than risk from the time of surgery.

### 2.4. Missing Data

Variables with substantial or systematically absent documentation were excluded before modelling. Retained predictors had minimal missingness. For model development and validation, missing predictor data were handled using multiple imputation by chained equations under a missing-at-random assumption, with five imputations performed separately in the development and temporal validation cohorts. Descriptive summaries and unadjusted between-group comparisons in Table 1 and Table S2 were based on observed data using available-case analysis.

### 2.5. Statistical Analysis

Continuous variables are reported as mean (standard deviation) or median (interquartile range) and categorical variables as number (percentage). Between-group differences were examined using the independent-samples *t*-test, Mann–Whitney U test, chi-square test, or Fisher exact test as appropriate. These comparisons were descriptive.

Model development used a two-stage strategy applied exclusively in the development cohort. Preoperative candidate predictors were reduced using LASSO logistic regression, with λ_min selected by 10-fold cross-validation. Variables retained by LASSO were entered into multivariable logistic regression to construct Model 1, with final inclusion guided by clinical and literature-based considerations rather than additional variable addition. Multicollinearity was evaluated using the variance inflation factor (VIF); all VIFs were below 3.

To construct Model 2, a 10-fold cross-fitting procedure generated an out-of-fold linear predictor (LP). In each iteration, Model 1 was fitted on 9 folds and applied to the held-out fold; this was repeated until each participant had an LP derived from a model in which their own outcome was not used. The LP summarises the aggregate preoperative risk as a continuous variable and was included alongside eligible intraoperative and early postoperative variables in a second LASSO procedure (λ_min), followed by multivariable logistic regression to construct Model 2. Individual preoperative variables already represented by the LP were not reconsidered separately.

For temporal validation, Model 1 was refitted to the complete development cohort and its fixed coefficients applied to validation-cohort patients to obtain Model 1 predicted probabilities. For each validation-cohort patient, the Model 1 LP was then computed and, together with the remaining Model 2 predictors, entered into the development-derived Model 2 equation. Neither model was recalibrated in the validation cohort.

Model performance was assessed using discrimination, calibration, overall accuracy (Brier score), and clinical utility (decision-curve analysis) [20,21]. AUCs were compared using DeLong’s test. Internal validation used 1000 bootstrap resamples that repeated the full modelling pipeline to estimate optimism-corrected performance. Continuous net reclassification improvement and integrated discrimination improvement were used as supplementary comparisons of Models 1 and 2 in both the development cohort and temporal validation cohorts and interpreted cautiously [22]. Among patients with ARDS, an exploratory model (Model 3) distinguishing moderate-to-severe from mild ARDS was developed using LASSO selection followed by multivariable logistic regression. Its performance was evaluated using the same metrics and internally validated using 1000 bootstrap resamples that repeated variable selection and coefficient estimation. Given the small sample and lack of independent validation, Model 3 was considered exploratory and unsuitable for individual prediction. Its variable-selection, regression, and performance results are reported in the Supplementary Material.

Analyses used R version 4.5.2. Tests were two-sided; *p* < 0.05 was considered statistically significant, with effect estimates and confidence intervals emphasised over isolated *p* values.

## 3. Results

### 3.1. Participants and Outcomes

Of 879 patients assessed, 283 were excluded and 596 were included: 395 in the development cohort and 201 in the temporal validation cohort (Figure 1). Characteristics and outcomes are presented for the development cohort; the validation dataset was restricted to model predictors and outcomes.

Alt text: Flowchart showing the selection of 596 patients from 879 assessed patients and their allocation to the development and temporal validation cohorts.

In the development cohort, 207 patients (52.4%) were male and the median age was 54 years (interquartile range, 45–61 years). Thymoma accounted for 70.6% of tumours; 83.5% of patients had Masaoka–Koga stage I–II disease. Selected characteristics and postoperative ARDS status stratified by sex are shown in Supplementary Table S1.

Postoperative ARDS occurred in 116 patients (29.4%): 53 (45.7%) had mild disease and 63 (54.3%) had moderate-to-severe disease. In the temporal validation cohort, 58 of 201 patients (28.9%) developed ARDS, a proportion that did not differ significantly from the development cohort (*p* = 0.972). Review of event timing showed that no documented ARDS events occurred before the postoperative landmark in either cohort (development cohort: 0 of 116 ARDS events; temporal validation cohort: 0 of 58 ARDS events). Thus, all documented ARDS events included in the Model 2 analysis occurred after the landmark.

### 3.2. Characteristics by ARDS Status

Compared with patients without ARDS, those with ARDS were older and more often had MG, heart disease, pulmonary inflammation, smoking history, thymic carcinoma, stage III–IV disease, open surgery and perioperative transfusion. They also had longer operative and OLV durations, greater blood loss, higher early postoperative CRP and lower albumin (Table 1). Additional clinical and laboratory comparisons are reported in Supplementary Table S2.

### 3.3. Model Development

The first LASSO procedure retained age, BMI, DLCO%, MG, pulmonary inflammation and smoking history at λ_min; these predictors were entered into multivariable logistic regression to construct Model 1. Cross-fitting was then used to generate the out-of-fold LP. The second LASSO procedure retained the LP, OLV duration, Masaoka–Koga stage, perioperative transfusion, and early postoperative glucose, albumin and CRP for Model 2. The LASSO coefficient profiles and cross-validation curves are shown in Supplementary Figures S1 and S2.

**Figure 2:**
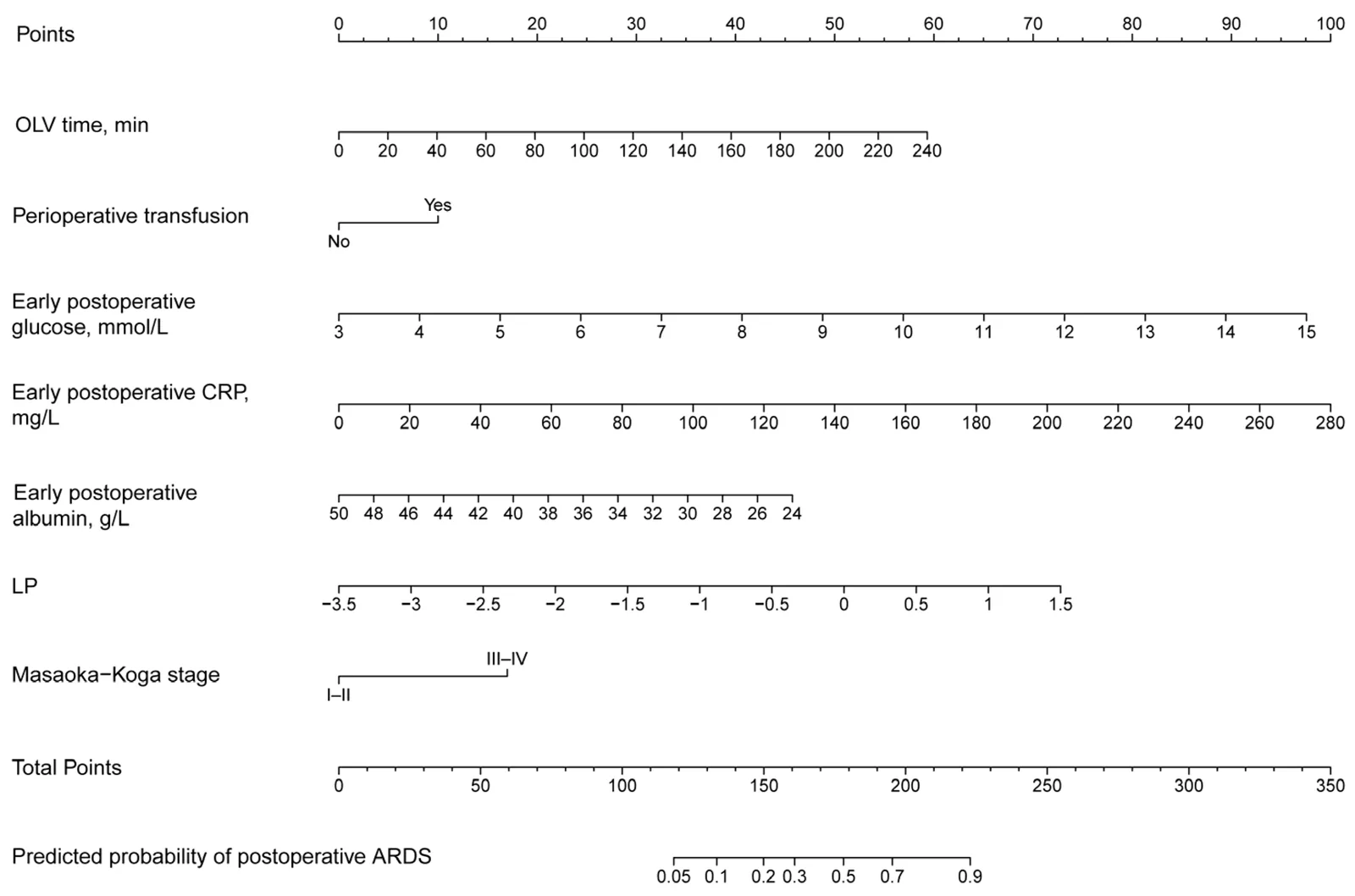
Nomogram for the postoperative landmark model. The nomogram estimates the probability of postoperative acute respiratory distress syndrome using LP, OLV time, perioperative transfusion, early postoperative glucose, albumin, CRP and Masaoka–Koga stage. Abbreviations: CRP, C-reactive protein; LP, linear predictor; OLV, one-lung ventilation. Alt text: Nomogram estimating postoperative acute respiratory distress syndrome probability from seven predictors and total points.

Multivariable regression estimates are presented in Table 2. In Model 1, age, BMI, MG, pulmonary inflammation, and smoking history were each associated with higher estimated odds of ARDS; DLCO% showed an inverse association that did not reach conventional significance (OR 0.984; *p* = 0.051). In Model 2, longer OLV duration, Masaoka–Koga stage III–IV, and higher early postoperative glucose and CRP were associated with higher odds of ARDS; albumin was inversely associated with the outcome. Perioperative transfusion was retained irrespective of its individual *p* value. The LP remained an independent predictor in Model 2 (OR 2.045; 95% CI 1.366–3.148; *p* < 0.001). A nomogram based on Model 2 is presented in Figure 2.

### 3.4. Model Performance

In the development cohort, the apparent AUC was 0.693 for Model 1 and 0.908 for Model 2 (difference 0.215; *p* < 0.001). After bootstrap optimism correction (1000 resamples, each repeating the full selection and cross-fitting pipeline), the Model 2 AUC was 0.898 (optimism 0.010). Model 2 had a lower Brier score (0.107 vs. 0.187; *p* < 0.001) and provided greater net benefit across most clinically relevant threshold probabilities. Continuous NRI was 1.152 and IDI was 0.391 (both *p* < 0.001), though these were calculated in the development data and should be interpreted cautiously.

**Figure 3:**
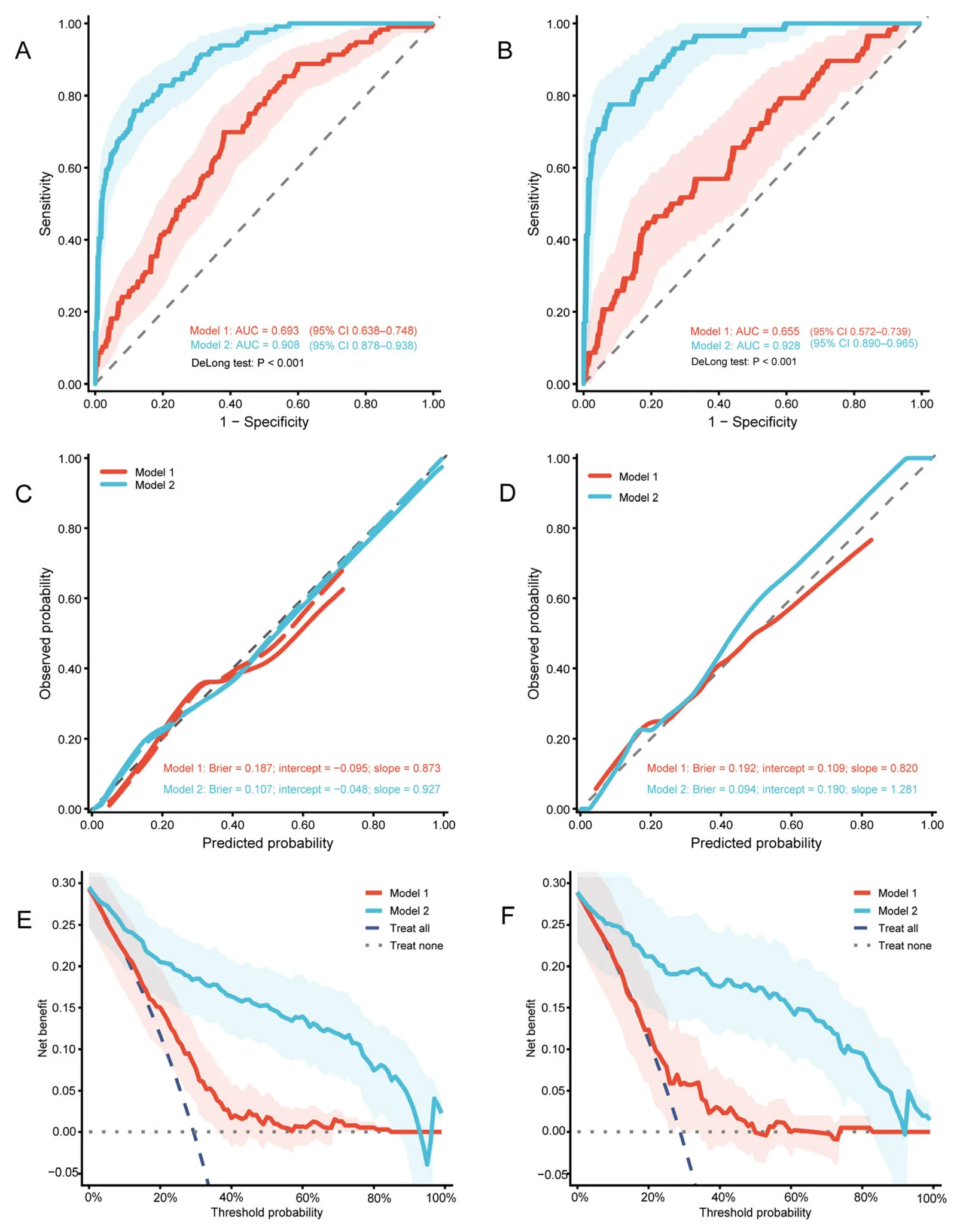
Performance of the preoperative and postoperative landmark models in the development and temporal validation cohorts. (**A**,**B**) Receiver operating characteristic curves; (**C**,**D**) calibration plots; and (**E**,**F**) decision curve analyses for the development and temporal validation cohorts, respectively. Model 2 showed better discrimination and clinical utility than Model 1 in both cohorts. In panel C, solid lines indicate bias-corrected calibration curves and dashed lines indicate apparent calibration curves. In panels C and D, the grey dashed diagonal line represents perfect calibration. Abbreviations: AUC, area under the receiver operating characteristic curve; CI, confidence interval. Alt text: Six-panel figure showing ROC curves, calibration plots and decision curves for Model 1 and Model 2 in the development and temporal validation cohorts.

In the temporal validation cohort, Model 2 had higher discrimination than Model 1 (AUC 0.928 vs. 0.655; *p* < 0.001) and a lower Brier score (0.094 vs. 0.192; *p* < 0.001). Model 2 calibration was close to the ideal line and DCA showed consistent net benefit. Model performance metrics are presented in Table 3. Receiver operating characteristic curves, calibration plots and decision curves for the development and temporal validation cohorts are shown in Figure 3.

### 3.5. Exploratory Severity Analysis

Among the 116 patients who developed postoperative ARDS, 53 had mild ARDS and 63 had moderate-to-severe ARDS. Compared with patients with mild ARDS, those with moderate-to-severe ARDS had lower MVV%, longer operative duration, greater blood loss, more frequent perioperative transfusion and higher early postoperative creatinine concentrations (Supplementary Table S3). LASSO selected MVV%, operative duration, and early postoperative creatinine, CRP and haemoglobin for inclusion in Model 3 (Supplementary Figure S3). None showed evidence of an independent association with ARDS severity after multivariable adjustment (Supplementary Table S4).

Model 3 had an apparent AUC of 0.747 (95% CI 0.657–0.837), a Brier score of 0.204 and a calibration slope of 0.759. The discrimination, calibration and decision curve analyses of Model 3 are shown in Supplementary Figure S4. Because these estimates were obtained in the same small subgroup used for model development, they are likely to be optimistic. Model 3 should therefore be regarded as hypothesis-generating and should not be used for individual prediction or clinical decision-making.

## 4. Discussion

### 4.1. Principal Findings

In this single-centre retrospective study, a staged postoperative landmark model incorporating intraoperative and early postoperative information substantially outperformed a preoperative-only model for predicting ARDS after TET surgery. The performance advantage was consistent across discrimination, calibration, net benefit, and temporal validation.

The observed ARDS proportion (29.4%) warrants cautious interpretation [7,23]. Beijing Chest Hospital is a specialist thoracic referral centre and receives patients with complex or advanced tumours who may carry a higher burden of perioperative risk than those reported in community or mixed institutional series. Additionally, ARDS was adjudicated retrospectively using the 2024 global definition, which accommodates a broader range of respiratory-support modalities and oxygenation thresholds than earlier criteria. The combined effect of case-mix enrichment and more inclusive diagnostic criteria is likely to inflate the observed proportion relative to estimates based on the Berlin definition in general surgical populations, and the figure should not be interpreted as a population incidence.

### 4.2. Interpretation of Model Predictors

The Model 1 predictors may reflect distinct but interrelated dimensions of preoperative vulnerability. Reduced DLCO% indicates impaired gas-exchange reserve and may increase susceptibility to oxygenation failure during OLV. Smoking and pulmonary inflammation may reflect existing airway or parenchymal injury that amplifies the inflammatory response to surgical stress [24]. MG impairs respiratory muscle function and airway clearance, predisposing patients to atelectasis and postoperative respiratory failure. Age and BMI may reflect differences in physiological reserve and metabolic–inflammatory status. These associations arise from an observational dataset and do not establish causal effects.

The incremental predictive value of Model 2 supports the concept that ARDS risk evolves from baseline susceptibility and perioperative exposure, allowing risk reassessment after additional perioperative information becomes available [17]. Prolonged OLV may expose the ventilated lung to mechanical stress and the collapsed lung to atelectatic and ischaemia–reperfusion injury [10,25]. Thoracic-surgery trials suggest that ventilation strategy can influence pulmonary outcomes, although effects vary by strategy and endpoint [9,26,27]. More broadly, an individual-patient-data meta-analysis of randomized surgical trials found fewer postoperative pulmonary complications with protective ventilation using lower tidal volumes [28]. Advanced Masaoka–Koga stage is associated with more extensive local invasion and greater operative exposure. Perioperative transfusion has been associated with postoperative ARDS, although confounding by bleeding and surgical complexity remains likely [29,30]. Early glucose, albumin and CRP should be interpreted as integrated markers of stress, protein–vascular balance [31,32] and inflammation rather than causal treatment targets; supporting evidence is indirect and uses different sampling windows or outcomes. Low albumin has also been incorporated as a predictor in a postoperative ARDS nomogram developed for patients undergoing surgical repair of type A acute aortic dissection [33]. These biomarkers were measured before the first clinical ARDS diagnosis and should be regarded as predictive markers rather than treatment targets; the present study does not demonstrate that their correction would reduce ARDS risk.

Patients undergoing TET surgery differ from general thoracic surgical populations in mediastinal anatomy, extent of resection, and frequency of MG, limiting direct extrapolation from other settings. Nevertheless, the associations involving pulmonary reserve, transfusion, and inflammatory markers broadly accord with findings reported for lung and oesophageal surgery.

### 4.3. Methodological Considerations

The cross-fitting approach reduced direct reuse of individual outcomes when generating the preoperative LP for Model 2. However, cross-fitting does not eliminate optimism arising from feature selection and coefficient estimation, particularly in a single-centre dataset of modest size [34,35]. Although same-centre temporal validation provides a stronger assessment than apparent performance alone, it does not establish geographical transportability. Independent centre-based validation has been used in multicentre postoperative ARDS prediction studies in other surgical populations [36]. Each bootstrap resample in the internal validation repeated the complete pipeline—including LASSO screening, cross-fitting, and model estimation—to produce estimates of optimism that account for these sources of variability. Performance estimates should accordingly be interpreted in conjunction with the optimism-corrected AUC and the temporal validation results. Model 3 was intentionally treated as an exploratory subgroup analysis. Its predictor selection, coefficient estimation and performance assessment were conducted in the same 116 patients, without temporal or external validation. The reported discrimination, calibration and decision curves therefore describe apparent performance only and should not be interpreted as evidence of clinical usefulness.

### 4.4. Temporal Drift and Long-Term Model Viability

The study spans more than two decades, during which surgical, anaesthetic, imaging, and other perioperative practices evolved. Detailed longitudinal information on specific institutional changes in surgical, anaesthetic, and imaging protocols was not consistently available across the entire study period; therefore, the effects of individual practice changes on model performance could not be quantified. Such changes may have affected ARDS ascertainment, the distribution of model predictors, and model performance over time. Although the temporal validation cohort provides some assessment of performance in a more recent clinical setting, same-centre temporal validation does not establish long-term model stability or geographical transportability [37,38]. Periodic reassessment of calibration and overall model performance, with recalibration or model updating when required, may therefore be necessary before and during future clinical implementation [39,40].

### 4.5. Study Limitations

This retrospective single-centre study is subject to selection and information bias and residual confounding, and the lack of independent external validation remains a key limitation. For some earlier cases, the temporal resolution of electronic documentation was less granular than in more recent records; therefore, the temporal ordering of final pathology availability and the first documented ARDS diagnosis was determined from the best available clinical documentation. Early postoperative predictors may partly reflect perioperative management or subclinical lung injury already present before overt ARDS. Multiple imputation cannot eliminate bias if data were not missing at random. Finally, Model 3 was developed and evaluated in the same small subgroup of patients with ARDS, including only 63 moderate-to-severe events, without independent validation; its performance estimates are therefore susceptible to overfitting and optimism and should be considered exploratory [35]. Because Model 2 incorporates pathological staging, its clinical application requires availability of postoperative pathological information and should be interpreted as a landmark reassessment model rather than an immediate postoperative prediction tool.

### 4.6. Recommendations for Future Research

Future studies should prospectively evaluate the staged prediction strategy and externally validate the models across multiple centres with different case mixes and perioperative practices. Further work should assess calibration and clinical utility over time and determine whether model recalibration or updating is required in different clinical settings. Prospective impact studies are also needed before routine clinical implementation.

## 5. Conclusions

A staged model incorporating intraoperative and early postoperative information predicted postoperative ARDS after TET surgery more accurately than a preoperative-only model in both the development and same-centre temporal validation cohorts. The model may support sequential perioperative risk assessment, but independent multicentre validation, potential model updating and prospective impact evaluation are required before clinical implementation. The exploratory severity model requires separate development and validation in a larger cohort and should not be used for individual prediction.

## Figures and Tables

**Table 1:** Selected characteristics of patients with and without postoperative acute respiratory distress syndrome in the development cohort.

Characteristic	Non-ARDS *N* = 279	ARDS *N* = 116	*p* Value ^a^
Age, years, Median (Q1, Q3)	53.0 (44.0, 60.0)	56.0 (48.0, 65.0)	<0.001
Sex, *n* (%)			0.047
Female	142 (50.9%)	46 (39.7%)	
Male	137 (49.1%)	70 (60.3%)	
BMI, kg/m^2^, Median (Q1, Q3)	24.2 (22.0, 26.7)	25.3 (23.0, 27.6)	0.023
Myasthenia gravis, *n* (%)			0.019
No	246 (88.2%)	91 (78.4%)	
Yes	33 (11.8%)	25 (21.6%)	
Hypertension, *n* (%)			0.294
No	213 (76.3%)	82 (70.7%)	
Yes	66 (23.7%)	34 (29.3%)	
Diabetes mellitus, *n* (%)			0.100
No	255 (91.4%)	99 (85.3%)	
Yes	24 (8.6%)	17 (14.7%)	
Heart disease, *n* (%)			0.007
No	264 (94.6%)	100 (86.2%)	
Yes	15 (5.4%)	16 (13.8%)	
Pulmonary inflammation, *n* (%)			0.024
No	201 (72.0%)	70 (60.3%)	
Yes	78 (28.0%)	46 (39.7%)	
Smoking history, *n* (%)			0.009
No	200 (71.7%)	67 (57.8%)	
Yes	79 (28.3%)	49 (42.2%)	
FEV_1_/FVC, Median (Q1, Q3)	97.5 (93.8, 103.7)	97.5 (92.2, 103.7)	0.525
MVV, % predicted, Median (Q1, Q3)	91.8 (80.4, 108.1)	91.8 (73.1, 102.7)	0.094
DLCO, % predicted, Median (Q1, Q3)	84.7 (78.3, 93.9)	84.7 (71.1, 91.7)	0.310
Tumour size, mm, Median (Q1, Q3)	5.0 (3.0, 6.0)	6.0 (3.5, 8.0)	0.002
Histological type, *n* (%)			<0.001
Thymoma	225 (80.6%)	54 (46.6%)	
Thymic carcinoma	54 (19.4%)	62 (53.4%)	
Masaoka–Koga stage, *n* (%)			<0.001
Stage I–II	247 (88.5%)	83 (71.6%)	
Stage III–IV	32 (11.5%)	33 (28.4%)	
Surgical approach, *n* (%)			<0.001
Minimally invasive	173 (62.0%)	50 (43.1%)	
Open thoracotomy	106 (38.0%)	66 (56.9%)	
Operative duration, min, Median (Q1, Q3)	107.0 (80.0, 150.0)	165.0 (117.5, 216.0)	<0.001
OLV time, min, Median (Q1, Q3)	60.0 (40.0, 80.0)	105.0 (60.0, 147.5)	<0.001
Intraoperative blood loss, mL, Median (Q1, Q3)	50.0 (20.0, 150.0)	120.0 (50.0, 500.0)	<0.001
Perioperative transfusion, *n* (%)			<0.001
No	257 (92.1%)	75 (64.7%)	
Yes	22 (7.9%)	41 (35.3%)	
Early postoperative glucose, mmol/L, Median (Q1, Q3)	6.3 (5.4, 7.3)	8.4 (7.2, 10.0)	<0.001
Early postoperative albumin, g/L, Median (Q1, Q3)	37.1 (34.8, 39.9)	34.7 (32.2, 37.8)	<0.001
Early postoperative CRP, mg/L, Median (Q1, Q3)	65.2 (38.6, 92.3)	149.4 (86.0, 197.0)	<0.001

^a.^ *p* values were calculated using the independent-samples *t*-test, Mann–Whitney U test, chi-square test, or Fisher exact test, as appropriate. Abbreviations: ARDS, acute respiratory distress syndrome; BMI, body mass index; CRP, C-reactive protein; DLCO, diffusing capacity of the lung for carbon monoxide; FEV_1_/FVC, forced expiratory volume in one second/forced vital capacity; MVV, maximal voluntary ventilation; OLV, one-lung ventilation. Continuous variables are presented as median (Q1, Q3), and categorical variables as number (percentage). Descriptive statistics and between-group comparisons were based on available observations using available-case analysis. Multiple imputation was used for model development and validation as described in the Methods.

**Table 2:** Multivariable logistic regression results for the preoperative and postoperative landmark models.

Variable	β	OR	95% CI	*p* Value
Results of multivariable logistic regression for Model 1				
Age	0.036	1.036	1.016, 1.058	<0.001
BMI	0.071	1.074	1.007, 1.146	0.031
DLCO, % predicted	−0.016	0.984	0.969, 1.000	0.051
Myasthenia gravis				
No (Ref.)	—	1.000	—	—
Yes	0.949	2.584	1.387, 4.813	0.003
Pulmonary inflammation				
No (Ref.)	—	1.000	—	—
Yes	0.510	1.666	1.027, 2.697	0.038
Smoking history				
No (Ref.)	—	1.000	—	—
Yes	0.641	1.898	1.179, 3.057	0.008
Results of multivariable logistic regression for Model 2				
LP	0.715	2.045	1.366, 3.148	<0.001
OLV time	0.012	1.012	1.006, 1.018	<0.001
Masaoka–Koga stage				
Stage I–II (Ref.)	—	1.000	—	—
Stage III–IV	0.834	2.303	1.067, 4.931	0.032
Perioperative transfusion				
No (Ref.)	—	1.000	—	—
Yes	0.491	1.634	0.711, 3.743	0.244
Early postoperative glucose	0.400	1.491	1.295, 1.735	<0.001
Early postoperative albumin	−0.086	0.917	0.846, 0.992	0.033
Early postoperative CRP	0.018	1.018	1.012, 1.024	<0.001

Abbreviations: BMI, body mass index; CI, confidence interval; CRP, C-reactive protein; DLCO, diffusing capacity of the lung for carbon monoxide; LP, linear predictor; OLV, one-lung ventilation; OR, odds ratio. Ref., reference category. Logistic regression coefficients (β), odds ratios (ORs), 95% confidence intervals (CIs), and *p* values are presented.

**Table 3:** Performance of the preoperative and postoperative landmark models in the development and temporal validation cohorts.

Metric	Model 1	Model 2
Development cohort		
Apparent AUC (95% CI)	0.693 (0.638–0.748)	0.908 (0.878 to 0.938)
Optimism-corrected AUC	0.668	0.898
Optimism	0.024	0.010
ΔAUC (Model 2 vs. Model 1)	—	0.215
DeLong test *p* value	—	<0.001
Continuous NRI (95% CI)	—	1.152 (0.959 to 1.335)
NRI *p* value	—	<0.001
IDI (95% CI)	—	0.391 (0.330 to 0.452)
IDI *p* value	—	<0.001
Brier score	0.187	0.107
ΔBrier score (95% CI)	—	−0.081 (−0.102 to −0.059)
ΔBrier *p* value	—	<0.001
Hosmer–Lemeshow *p* value	0.748	0.487
Temporal validation cohort		
AUC (95% CI)	0.655 (0.572–0.739)	0.928 (0.890 to 0.965)
ΔAUC (Model 2 vs. Model 1)	—	0.272
DeLong test *p* value	—	<0.001
Continuous NRI (95% CI)	—	1.217 (0.960 to 1.466)
NRI *p* value	—	<0.001
IDI (95% CI)	—	0.418 (0.332 to 0.503)
IDI *p* value	—	<0.001
Discrimination slope	0.078	0.496
Brier score	0.192	0.094
ΔBrier score (95% CI)	—	−0.099 (−0.128 to −0.069)
ΔBrier *p* value	—	<0.001
Hosmer–Lemeshow *p* value	0.821	0.598

Abbreviations: AUC, area under the receiver operating characteristic curve; CI, confidence interval; IDI, integrated discrimination improvement; NRI, net reclassification improvement. Model performance was assessed using discrimination, calibration, and reclassification metrics. Higher AUC and lower Brier score indicate better performance.

## Data Availability

The data presented in this study are not publicly available due to patient privacy and institutional data protection policies but are available from the corresponding author on reasonable request.

## Supplementary Materials

The following supporting information can be downloaded at https://www.mdpi.com/article/10.3390/jcm15197715/s1. Figure S1: LASSO analysis for the preoperative model; Figure S2: LASSO analysis for the postoperative landmark model; Figure S3: LASSO analysis for the exploratory severity model; Figure S4: Performance of the exploratory severity model; Table S1: Selected characteristics and postoperative acute respiratory distress syndrome status in the development cohort, stratified by sex; Table S2: Additional clinical and laboratory characteristics according to postoperative acute respiratory distress syndrome status in the development cohort; Table S3: Characteristics of patients with mild and moderate-to-severe acute respiratory distress syndrome in the development cohort; Table S4: Multivariable logistic regression results for the exploratory severity model. STROBE checklist [18]; TRIPOD+AI checklist [19,41].
