# Supplementary material for: Development and Temporal Validation of Staged Prediction Models for Acute Respiratory Distress Syndrome After Thymic Epithelial Tumour Resection
Source: J Clin Med. 2026 Oct 6;15(19):7715. doi: 10.3390/jcm15197715 (PMC13666171; doi:10.3390/jcm15197715)
Supplement: Supplementary file 1 [file jcm-15-07715-s001.zip › supplement-jcm.pdf]

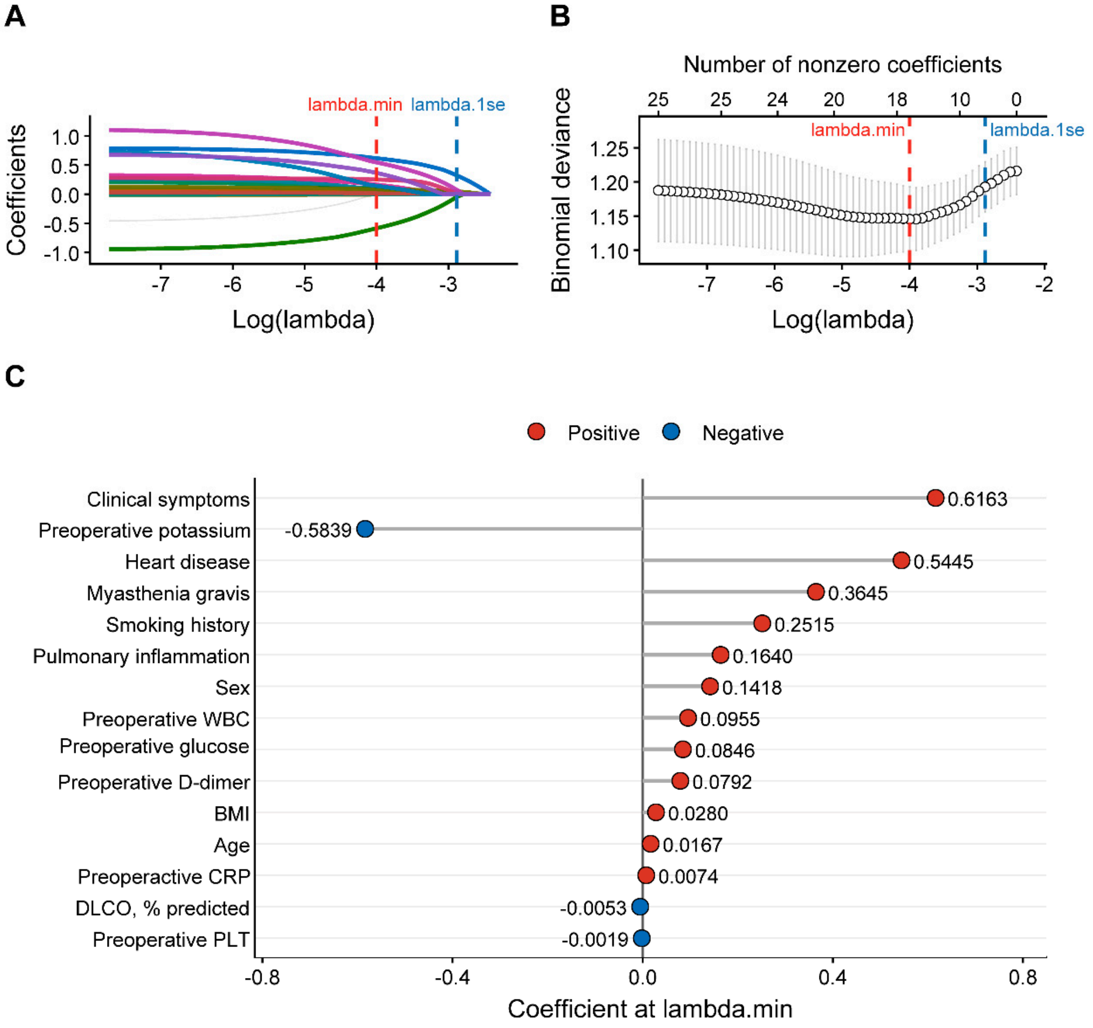

Supplementary Figure S1. LASSO analysis for the preoperative model. (A) Coefficient paths; (B) 10-fold cross-validation curve; and (C) coefficients at λ_min. Dashed lines indicate λ_min and λ_1se. Abbreviations: BMI, body mass index; CRP, C-reactive protein; DLCO, diffusing capacity of the lung for carbon monoxide; LASSO, least absolute shrinkage and selection operator; PLT, platelet count; WBC, white blood cell count.

Alt text: Three-panel LASSO analysis showing coefficient paths, cross-validation and coefficients at λ_min for the preoperative model.

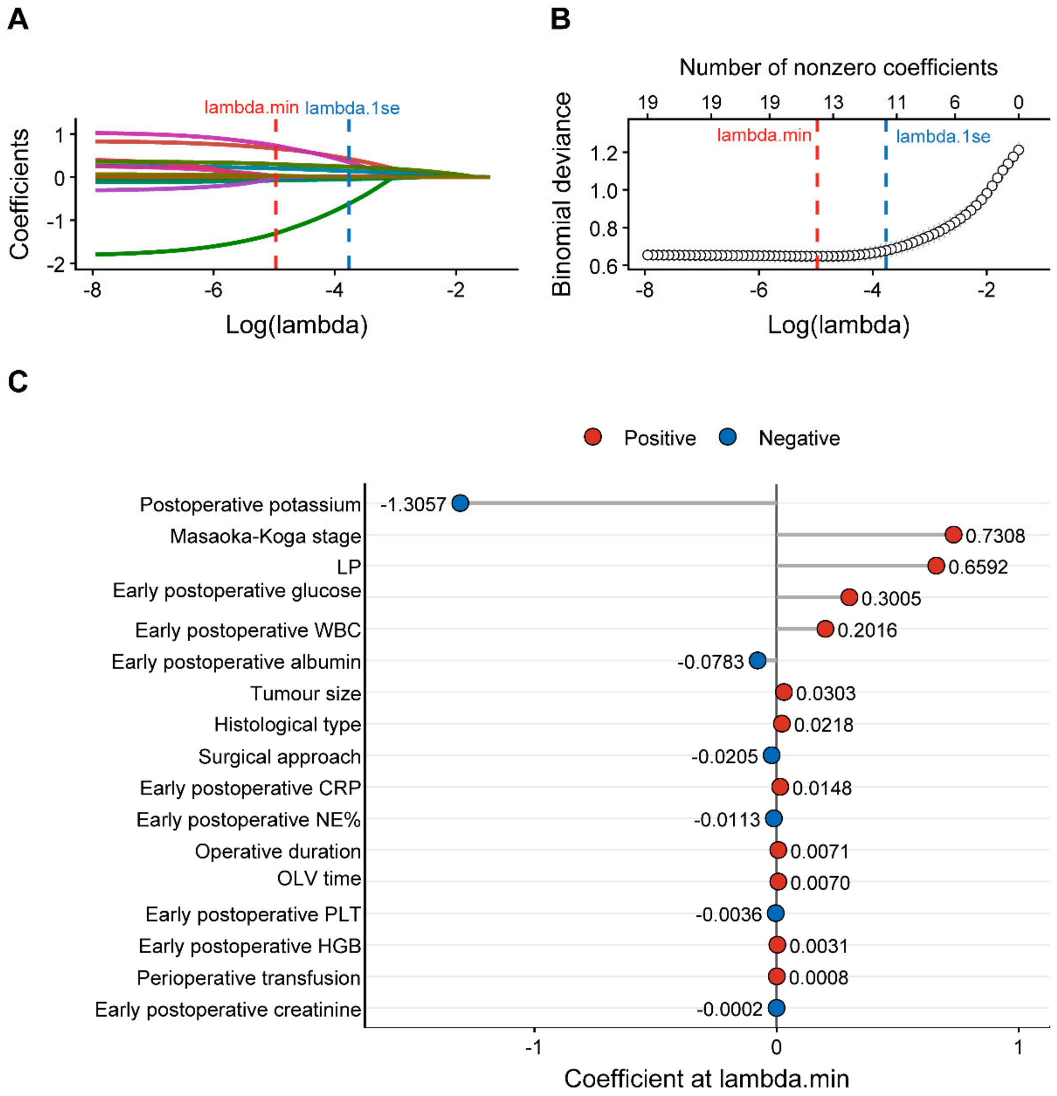

Supplementary Figure S2. LASSO analysis for the postoperative landmark model. (A) Coefficient paths; (B) 10-fold cross-validation curve; and (C) coefficients at λ_min. Dashed lines indicate λ_min and λ_1se.Abbreviations: CRP, C-reactive protein; HGB, haemoglobin; K, potassium; LASSO, least absolute shrinkage and selection operator; LP, linear predictor; NE, neutrophil percentage; OLV, one-lung ventilation; PLT, platelet count; WBC, white blood cell count; WHO, World Health Organization.

Alt text: Three-panel LASSO analysis showing coefficient paths, cross-validation and coefficients at λ_min for the postoperative landmark model.

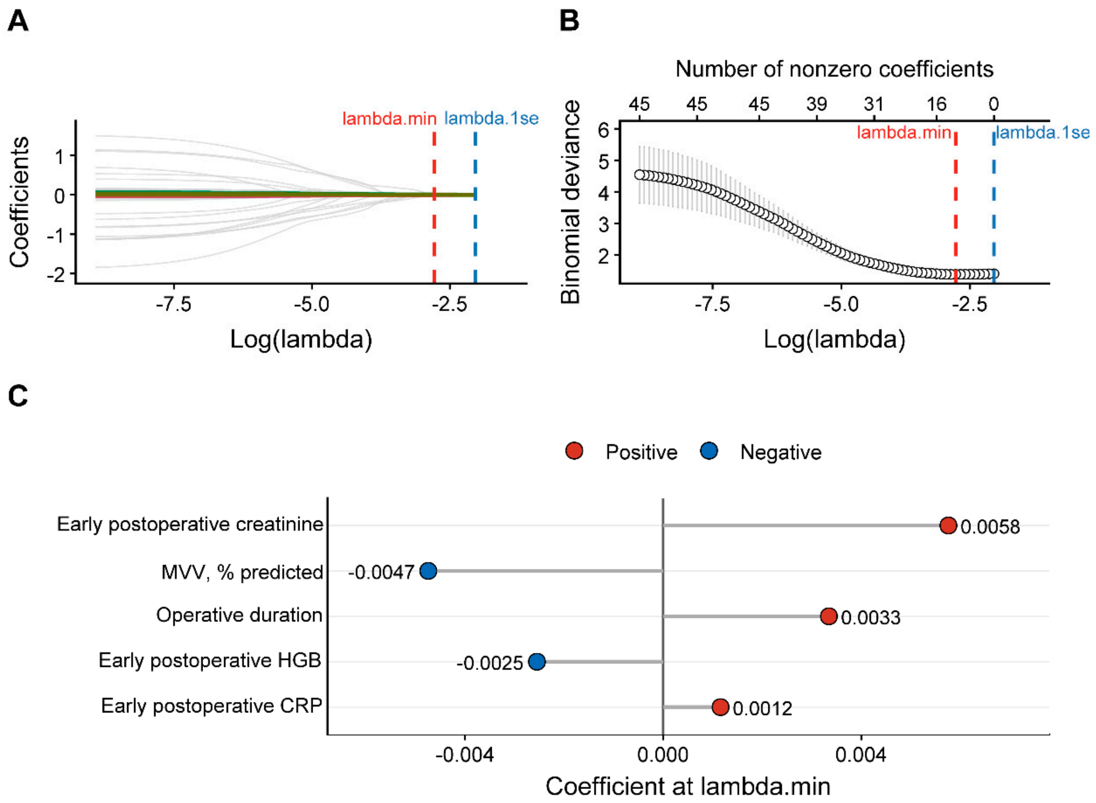

Supplementary Figure S3. LASSO analysis for the exploratory severity model. (A) Coefficient paths; (B) 10-fold cross-validation curve; and (C) coefficients at λ_min. Dashed lines indicate λ_min and λ_1se.Abbreviations: CRP, C-reactive protein; HGB, haemoglobin; LASSO, least absolute shrinkage and selection operator; MVV, maximal voluntary ventilation.

Alt text: Three-panel LASSO analysis for the exploratory model distinguishing moderate-to-severe from mild postoperative acute respiratory distress syndrome.

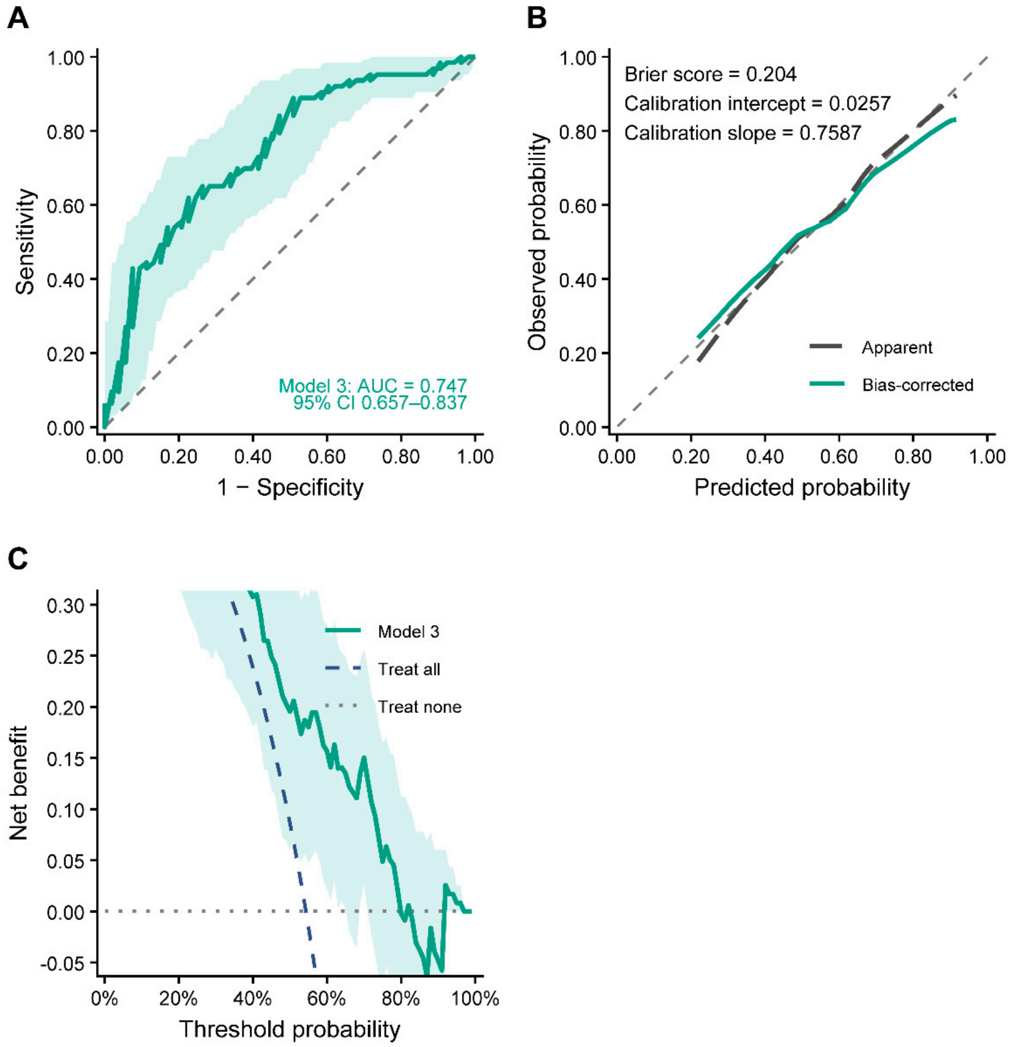

Supplementary Figure S4. Performance of the exploratory severity model. (A) Receiver operating characteristic curve; (B) apparent and bias-corrected calibration curves; and (C) decision curve analysis. The apparent AUC was 0.747, the Brier score was 0.204 and the calibration slope was 0.759.

Abbreviations: AUC, area under the receiver operating characteristic curve; DCA, decision curve analysis; ROC, receiver operating characteristic.

Alt text: Three-panel figure showing discrimination, calibration and clinical net benefit of the exploratory severity model.

Supplementary Table S1. Selected characteristics and postoperative acute respiratory distress syndrome status in the development cohort, stratified by sex

Characteristic	Female<br>*N* = 188	Male<br>*N* = 207
Age, years, Median (Q1, Q3)	55.5 (46.0, 62.0)	53.0 (44.0, 60.0)
Body mass index, kg/m², Median (Q1, Q3)	24.2 (21.7, 26.9)	24.8 (22.8, 27.0)
Myasthenia gravis, *n* (%)		
No	161 (85.6%)	176 (85.0%)
Yes	27 (14.4%)	31 (15.0%)
Hypertension, *n* (%)		
No	142 (75.5%)	153 (73.9%)
Yes	46 (24.5%)	54 (26.1%)
Diabetes mellitus, *n* (%)		
No	168 (89.4%)	186 (89.9%)
Yes	20 (10.6%)	21 (10.1%)
Heart disease, *n* (%)		
No	178 (94.7%)	186 (89.9%)
Yes	10 (5.3%)	21 (10.1%)
Pulmonary inflammation, *n* (%)		
No	133 (70.7%)	138 (66.7%)
Yes	55 (29.3%)	69 (33.3%)
Smoking history, *n* (%)		
No	179 (95.2%)	88 (42.5%)
Yes	9 (4.8%)	119 (57.5%)
FEV_1/FVC, Median (Q1, Q3)	97.8 (94.7, 104.1)	97.5 (92.2, 103.5)
MVV, % predicted, Median (Q1, Q3)	91.8 (73.3, 103.1)	91.8 (82.0, 110.8)
DLCO, % predicted, Median (Q1, Q3)	84.7 (75.8, 91.2)	84.7 (80.8, 96.1)
Tumour size, mm, Median (Q1, Q3)	5.0 (3.5, 7.0)	5.0 (3.0, 7.0)

Characteristic	Female<br>*N* = 188	Male<br>*N* = 207
Histological type, *n* (%)		
Thymoma	142 (75.5%)	137 (66.2%)
Thymic carcinoma	46 (24.5%)	70 (33.8%)
Masaoka-Koga stage, *n* (%)		
Stage I–II	167 (88.8%)	163 (78.7%)
Stage III–IV	21 (11.2%)	44 (21.3%)
Surgical approach, *n* (%)		
Minimally invasive	114 (60.6%)	109 (52.7%)
Open thoracotomy	74 (39.4%)	98 (47.3%)
Operative time, min, Median (Q1, Q3)	120.0 (80.0, 160.0)	125.0 (92.0, 176.0)
One-lung ventilation time, min, Median (Q1, Q3)	60.0 (35.0, 95.0)	70.0 (45.0, 110.0)
Intraoperative blood loss, mL, Median (Q1, Q3)	50.0 (20.0, 200.0)	100.0 (20.0, 300.0)
Perioperative transfusion, *n* (%)		
No	165 (87.8%)	167 (80.7%)
Yes	23 (12.2%)	40 (19.3%)
Early postoperative glucose, mmol/L, Median (Q1, Q3)	6.8 (5.7, 8.6)	6.7 (5.7, 7.9)
Early postoperative albumin, g/L, Mean ± SD	35.9 ± 3.9	37.2 ± 4.1
Early postoperative CRP, mg/L, Median (Q1, Q3)	69.4 (38.7, 102.4)	92.3 (55.3, 140.2)
Postoperative ARDS, *n* (%)		
No	142 (75.5%)	137 (66.2%)
Yes	46 (24.5%)	70 (33.8%)

Abbreviations: ARDS, acute respiratory distress syndrome; BMI, body mass index; CRP, C-reactive protein; DLCO, diffusing capacity of the lung for carbon monoxide; FEV_1/FVC, forced expiratory volume in one second/forced vital capacity; MVV, maximal voluntary ventilation. Continuous variables are presented as median (Q1, Q3) or mean ± SD, as indicated. Categorical variables are presented as number (percentage).

Supplementary Table S2. Additional clinical and laboratory characteristics according to postoperative acute respiratory distress syndrome status in the development cohort

Characteristic	Non-ARDS<br>*N* = 279	ARDS<br>*N* = 116	*p* value[a]
Clinical symptoms, *n* (%)			<0.001
No	168 (60.2%)	45 (38.8%)	
Yes	111 (39.8%)	71 (61.2%)	
Preoperative glucose, mmol/L, Median (Q1, Q3)	5.8 (5.0, 7.2)	6.4 (5.2, 8.0)	0.003
Preoperative sodium, mmol/L, Median (Q1, Q3)	138.8 (136.0, 140.9)	138 (136.0, 140.0)	0.262
Preoperative potassium, mmol/L, Median (Q1, Q3)	3.8 (3.6, 4.0)	3.7 (3.5, 3.9)	0.012
Preoperative creatinine, μmol/L, Mean ± SD	70.1 ± 14.2	70.1 ± 15.3	0.997
Preoperative albumin, g/L, Mean ± SD	41.4 ± 3.7	41 ± 3.7	0.420
Preoperative CRP, mg/L, Median (Q1, Q3)	1.5 (0.6, 5.3)	2.6 (1.1, 5.3)	0.007
Preoperative WBC, $\times10^9$/L, Median (Q1, Q3)	5.9 (5.0, 7.0)	6.3 (5.3, 7.6)	0.027
Preoperative NE%, %, Mean ± SD	57.7 ± 9.3	58.6 ± 9.9	0.625
Preoperative HGB, g/L, Mean ± SD	134.0 ± 16.9	135.5 ± 16.2	0.567
Preoperative PLT, $\times10^9$/L, Median (Q1, Q3)	230.0 (189.0, 269.0)	225 (186.5, 271.2)	0.397
Preoperative APTT, s, Median (Q1, Q3)	31.0 (27.5, 33.4)	30.3 (26.8, 33.5)	0.466
Preoperative PT, s, Median (Q1, Q3)	11.5 (10.9, 12.2)	11.4 (10.7, 12.3)	0.941
Preoperative FIB, mg/dL, Median (Q1, Q3)	2.9 (2.4, 3.6)	3.2 (2.8, 3.7)	0.003
Preoperative D-dimer, mg/L, Median (Q1, Q3)	0.2 (0.2, 0.4)	0.3 (0.2, 0.5)	0.006
Early postoperative sodium, mmol/L, Mean ± SD	138.7 ± 3.1	138.6 ± 3.3	0.702
Early postoperative potassium, mmol/L, Mean ± SD	3.9 ± 0.4	3.8 ± 0.4	0.064
Early postoperative creatinine, μmol/L, Median (Q1, Q3)	65.5 (54.6, 74.7)	67.5 (57.9, 79.5)	0.076

Characteristic	Non-ARDS<br>*N* = 279	ARDS<br>*N* = 116	*p* value[a]
Early postoperative WBC, $\times 10^9$/L, Median (Q1, Q3)	12.3 (10.3, 13.8)	15.4 (12.9, 19.1)	<0.001
Early postoperative NE%, %, Median (Q1, Q3)	85.5 (81.5, 88.8)	87.0 (83.6, 90.2)	0.010
Early postoperative HGB, g/L, Mean ± SD	127.6 ± 17.4	123.7 ± 21.3	0.082
Early postoperative PLT, $\times 10^9$/L, Median (Q1, Q3)	211.0 (174.0, 253.0)	191.5 (153.8, 249.0)	0.024

a. *p* values were calculated using the independent-samples t-test, Mann–Whitney U test, chi-square test or Fisher's exact test, as appropriate. Abbreviations: ARDS, acute respiratory distress syndrome; APTT, activated partial thromboplastin time; CRP, C-reactive protein; FIB, fibrinogen; HGB, haemoglobin; NE%, neutrophil percentage; PLT, platelet count; PT, prothrombin time; WBC, white blood cell count. Continuous variables are presented as median (Q1, Q3) or mean ± SD, as indicated. Categorical variables are presented as number (percentage).

Supplementary Table S3. Characteristics of patients with mild and moderate-to-severe acute respiratory distress syndrome in the development cohort

Characteristic	Mild ARDS<br>$N = 53$	Moderate-to-severe ARDS<br>$N = 63$	p value[a]
Age, years, Median (Q1, Q3)	56.0 (49.0, 65.0)	55.0 (48.0, 65.0)	0.794
Sex, n (%)			0.708
Female	20 (37.7%)	26 (41.3%)	
Male	33 (62.3%)	37 (58.7%)	
Body mass index, kg/m², Median (Q1, Q3)	24.4 (22.3, 27.0)	26.1 (23.6, 27.7)	0.094
Smoking history, n (%)			0.706
No	32 (60.4%)	35 (55.6%)	
Yes	21 (39.6%)	28 (44.4%)	
Myasthenia gravis, n (%)			0.651
No	43 (81.1%)	48 (76.2%)	
Yes	10 (18.9%)	15 (23.8%)	
Hypertension, n (%)			0.547
No	39 (73.6%)	43 (68.3%)	
Yes	14 (26.4%)	20 (31.7%)	
Diabetes mellitus, n (%)			1.000
No	45 (84.9%)	54 (85.7%)	
Yes	8 (15.1%)	9 (14.3%)	
Heart disease, n (%)			0.283
No	48 (90.6%)	52 (82.5%)	
Yes	5 (9.4%)	11 (17.5%)	
Pulmonary inflammation, n (%)			0.568
No	30 (56.6%)	40 (63.5%)	
Yes	23 (43.4%)	23 (36.5%)	

Characteristic	Mild ARDS<br>$N = 53$	Moderate-to-severe ARDS<br>$N = 63$	p value[a]
FEV_1/FVC, Median (Q1, Q3)	97.5 (92.3, 102.9)	98.6 (91.4, 103.7)	1.000
MVV, % predicted, Median (Q1, Q3)	91.8 (84.1, 110.0)	84.2 (61.0, 101.4)	0.026
DLCO, % predicted, Median (Q1, Q3)	84.7 (81.4, 91.8)	84.3 (69.3, 91.3)	0.252
Preoperative glucose, mmol/L, Median (Q1, Q3)	6.5 (5.5, 8.1)	6.0 (5.2, 7.8)	0.340
Preoperative sodium, mmol/L, Median (Q1, Q3)	138.0 (136.0, 139.5)	138.0 (136.0, 141.0)	0.301
Preoperative potassium, mmol/L, Median (Q1, Q3)	3.7 (3.4, 3.9)	3.7 (3.5, 3.9)	0.665
Preoperative creatinine, μmol/L, Mean ± SD	67.3 ± 14.1	72.5 ± 16.2	0.026
Preoperative albumin, g/L, Mean ± SD	41.2 ± 3.5	40.9 ± 3.8	0.780
Preoperative CRP, mg/L, Median (Q1, Q3)	1.8 (1.0, 5.3)	3.2 (1.2, 5.3)	0.331
Preoperative WBC, $\times 10^9$/L, Median (Q1, Q3)	6.2 (5.3, 7.1)	6.5 (5.3, 7.9)	0.444
Preoperative NE%, %, Mean ± SD	59.0 ± 10.1	58.3 ± 9.8	0.622
Preoperative HGB, g/L, Mean ± SD	136.8 ± 16.1	134.4 ± 16.3	0.487
Preoperative PLT, $\times 10^9$/L, Median (Q1, Q3)	232.0 (195.0, 274.0)	220.0 (174.5, 257.0)	0.347
Preoperative APTT, s, Median (Q1, Q3)	30.3 (26.5, 33.5)	30.4 (27.6, 33.3)	0.572
Preoperative PT, s, Median (Q1, Q3)	11.6 (10.9, 12.3)	11.4 (10.6, 12.4)	0.347
Preoperative FIB, mg/dL, Median (Q1, Q3)	3.2 (2.9, 3.7)	3.2 (2.7, 3.8)	0.504
Preoperative D-dimer, mg/L, Median (Q1, Q3)	0.3 (0.2, 0.5)	0.3 (0.2, 0.5)	0.790
Clinical symptoms, *n* (%)			0.251
No	24 (45.3%)	21 (33.3%)	
Yes	29 (54.7%)	42 (66.7%)	
Tumour size, mm, Median (Q1, Q3)	5.0 (2.6, 7.0)	6.0 (4.0, 8.5)	0.055
Histological type, *n* (%)			0.062
Thymoma	30 (56.6%)	24 (38.1%)	

Characteristic	Mild ARDS<br>*N* = 53	Moderate-to-severe ARDS<br>*N* = 63	*p* value[a]
Thymic carcinoma	23 (43.4%)	39 (61.9%)	
Masaoka–Koga stage, *n* (%)			0.685
Stage I–II	39 (73.6%)	44 (69.8%)	
Stage III–IV	14 (26.4%)	19 (30.2%)	
Surgical approach, *n* (%)			0.135
Minimally invasive	27 (50.9%)	23 (36.5%)	
Open thoracotomy	26 (49.1%)	40 (63.5%)	
Operative duration, min, Median (Q1, Q3)	147.0 (100.0, 198.0)	174.0 (135.0, 222.0)	0.008
OLV time, min, Median (Q1, Q3)	105.0 (70.0, 140.0)	110.0 (42.5, 152.5)	0.984
Intraoperative blood loss, mL, Median (Q1, Q3)	100.0 (50.0, 350.0)	200.0 (100.0, 700.0)	0.013
Perioperative transfusion, *n* (%)			0.032
No	40 (75.5%)	35 (55.6%)	
Yes	13 (24.5%)	28 (44.4%)	
Early postoperative glucose, mmol/L, Median (Q1, Q3)	8.4 (7.0, 9.8)	8.4 (7.2, 9.9)	0.600
Early postoperative sodium, mmol/L, Median (Q1, Q3)	138.0 (136.0, 140.0)	139.0 (136.0, 141.8)	0.105
Early postoperative potassium, mmol/L, Median (Q1, Q3)	3.8 (3.6, 4.1)	3.8 (3.5, 4.1)	0.998
Early postoperative creatinine, μmol/L, Median (Q1, Q3)	62.0 (55.0, 73.3)	72.8 (62.0, 86.0)	0.003
Early postoperative albumin, g/L, Mean ± SD	35.1 ± 4.1	34.7 ± 4.6	0.195
Early postoperative CRP, mg/L, Median (Q1, Q3)	114.2 (77.8, 184.4)	164.4 (94.5, 205.9)	0.052

Characteristic	Mild ARDS<br>*N* = 53	Moderate-to-severe ARDS<br>*N* = 63	*p* value[a]
Early postoperative WBC, ×10^9/L, Median (Q1, Q3)	15.1 (12.9, 18.6)	15.9 (12.8, 19.6)	0.872
Early postoperative NE%, %, Median (Q1, Q3)	87.1 (83.8, 90.5)	86.9 (82.6, 90.1)	0.855
Early postoperative HGB, g/L, Mean ± SD	127.7 ± 23.1	120.0 ± 19.9	0.025
Early postoperative PLT, ×10^9/L, Median (Q1, Q3)	205.0 (165.0, 251.0)	177.0 (139.0, 246.0)	0.105

a. *p* values were calculated using the independent-samples t-test, Mann–Whitney U test, chi-square test or Fisher's exact test, as appropriate. Abbreviations: APTT, activated partial thromboplastin time; ARDS, acute respiratory distress syndrome; BMI, body mass index; CRP, C-reactive protein; DLCO, diffusing capacity of the lung for carbon monoxide; FEV_1/FVC, forced expiratory volume in one second/forced vital capacity; FIB, fibrinogen; HGB, haemoglobin; MVV, maximal voluntary ventilation; NE%, neutrophil percentage; OLV, one-lung ventilation; PLT, platelet count; PT, prothrombin time; WBC, white blood cell count. Continuous variables are presented as median (Q1, Q3) or mean ± SD, as indicated. Categorical variables are presented as number (percentage).

Supplementary Table S4. Multivariable logistic regression results for the exploratory severity model

Variable	β	OR	95% CI	*P* value
MVV, % predicted	−0.015	0.985	0.968, 1.001	0.074
Operative duration, min	0.006	1.006	1.000, 1.013	0.060
Early postoperative creatinine, μmol/L	0.017	1.018	0.999, 1.041	0.091
Early postoperative HGB, g/L	−0.015	0.985	0.965, 1.004	0.135
Early postoperative CRP, mg/L	0.005	1.005	0.999, 1.011	0.102

The outcome was moderate-to-severe ARDS, with mild ARDS as the reference category. Odds ratios are expressed per 1-unit increase in each continuous predictor. Abbreviations: CI, confidence interval; CRP, C-reactive protein; HGB, haemoglobin; MVV, maximal voluntary ventilation; OR, odds ratio. Logistic regression coefficients (β), odds ratios (ORs), 95% confidence intervals (CIs), and P values are presented.
